#  Multi-walled Сarbon Nanotubes Penetrate into Plant Cells and Affect the Growth of Onobrychis arenaria Seedlings 

**Published:** 2011

**Authors:** E.A. Smirnova, A.A. Gusev, O.N. Zaitseva, E.M. Lazareva, G.E. Onishchenko, E.V. Kuznetsova, A.G. Tkachev, A.V. Feofanov, M.P. Kirpichnikov

**Affiliations:** Biology Faculty, Lomonosov Moscow State University; Derzhavin Tambov State University; Siberian Institute of Plant Physiology and Biochemistry, Siberian Branch, Russian Academy of Sciences; NanoTechCenter Ltd.; Shemyakin and Ovchinnikov Institute of Bioorganic Chemistry, Russian Academy of Sciences

**Keywords:** multi-walled carbon nanotubes, light microscopy, electron microscopy, electron diffraction pattern, *O. arenaria*seedlings

## Abstract

Engineered nanoparticles (ENPs) are now being used in many sectors of industry; however, the impact of ENPs on the environment still requires further study, since their use, recycling, and accidental spill can result in the accumulation of nanoparticles in the atmosphere, soil, and water. Plants are an integral part of ecosystems; hence their interaction with ENPs is inevitable. It is important to understand the consequences of this interaction and assess its potential effects. The present research is focused on studying the effects of the industrial material Taunit, containing multi-walled carbon nanotubes (MWNTs), on plants, and testing of its ability to penetrate into plant cells and tissues. Taunit has been found to stimulate the growth of roots and stems and cause an increase in peroxidase activity in*Onobrychis arenaria*seedlings. Peroxidase activity increases with decreasing concentration of Taunit from 1,000 to 100 mg/l. MWNTs from Taunit were detected in the cells and tissues of seedling roots and leaves, implying the ability of MWNTs to penetrate into roots and accumulate there, as well as their ability to be transported into seedling leaves. Thus, the changes in the physiological parameters of plants are associated not only with MWNT adsorption on the root surface, as previously believed, but also with their penetration, uptake and accumulation in the plant cells and tissues.

##  INTRODUCTION 

The great benefits of using nanomaterials in modern technologies are no longer questioned. However, the potential negative effects associated with the propagation and accumulation of nanomaterial components, such as nano-particles and nanofibers in the environment, require further study [1, 2]. Plants are the major components of ecosystems; subsequently, significant attention should be paid to the effects of various technogenical materials upon them [3–5]. Carbon nanomaterials (CNM), such as fullerenes, multi-walled carbon nanotubes (MWNTs), and single-walled carbon nanotubes (CWNTs), are a matter of special interest, as their industrial production is rapidly developing. Since nanotubes have a fibrillar form, they are compared with asbestos [6]. In light of such an analogy, potential adverse effects on living organisms can be anticipated [7]. Preliminary studies have provided evidence that MWNTs and SWNTs are pathogenic to animals [8], yet they have different effects on plants. MWNTs were shown to considerably increase the growth rate of tomato seedlings [9], have no effect on the growth parameters of wheat [10], and inhibit the growth of rice seedlings [11]. SWNTs have been shown to suppress the growth of tomato roots, but stimulate the root growth of onion and cucumber [12]. In contrast, MWNTs have a toxic effect on *Arabidopsis* cultured cells. [13]. High adsorption of MWNT/SWNT on the roots of seedlings was observed in all the experiments conducted. However, the penetration, uptake and accumulation of MWNTs/SWNTs in plant cells and tissues are not well documented . Furthermore, the mechanism of the development of the physiological changes caused by the exposure of plants to nanotubes also remains unclear. The present work was aimed at studying the effect of the industrial nanomaterial Taunit, containing MWNTs, on *O. arenaria* seedlings, and the ability of MWNTs to penetrate and accumulate in plant cells and tissues.

##  EXPERIMENTAL PART 

**Object of the study**

The object of the present study is industrial CNM Taunit (NanoTechCenter Ltd., Tambov, Russia). This material is a loose black powder, composed of grainy agglomerates with a size of several micrometers. Agglomerates mostly consist of entangled MWNT bundles. MWNTs have a hollow cylindrical structure; at least 2 µm long, with an external diameter of 20–70 nm and an internal diameter of 5–10 nm. Taunit is produced by chemical vapor deposition; its purity is above 98% [14].

**Seed germination and morphometric assessment**

The seeds of *Onobrychis arenaria* were germinated in a medium containing a colloidal aqueous solution of CNM Taunit with a concentration of 100 or 1,000 mg/l. Prior to use, CNM was dispersed in distilled water by ultrasonic treatment . Distilled water was used to prepare the control medium. The seeds (50 seeds per dish) were grown for 10 days on filter paper in glass Petri dishes (diameter of 90 mm) with 5 ml of a CNM suspension added. 200 seeds were used in each experiment. The growth conditions followed the requirements of the State Standard procedure GOST 12038-84 (Agricultural seeds. Methods for evaluation of germination ). The effect of CNM on esparcet seedlings was estimated on the basis of the following parameters: the rate of seed germination (%), the energy of germination (%), and the length of the roots and stems . The energy of germination and the rate of germination were determined as the ratio between the number of germinated seeds and the number of plated seeds by day 5 and day 10, respectively (%, in accordance with the State Standard).

**Extraction of soluble peroxidases and determination of their activity**

The weighed samples (2 g) of *O. arenaria * seedlings tissues were placed into 5 ml of a cold phosphate/citrate buffer (1 M solution of citric acid + 1 M NaH _2_ PO _4_ , two solutions combined to achieve pH 5.5) and ground in a porcelain mortar at 4°С [15]. The homogenate was centrifugated at 3,000 g for 15 min. The cleared supernatant was used to determine the activity of soluble peroxidases on the basis of the change rate (time, s) of the optical density at a wavelength of 580 nm in the reaction mixture containing 0.5 ml of 0.1 M solution of the phosphate/citrate buffer (pH 5.5), 0.5 ml of 0.3% Н _2_ О _2_ , 0.5 ml of 0.05% guaiacol (Sigma, USA), and 0.5 ml of the sample. Peroxidase activity was measured at 25 ^о^ С immediately after the enzymes were extracted from the samples. Enzymatic activity was calculated by Boyarkin’s method [16] and expressed in arbitrary units of activity per gram of fresh tissue weight per second, according to the following formula:

*А* = (ε × α × β × γ)/( *d * × *t* ),

 where ε is the extinction coefficient, 

 α is the ratio between the amount of buffer taken for extract preparation (ml) to fresh tissue weight (g), 

 β is the degree of additional dilution of the extract in the reaction mixture, 

 γ is the degree of constant dilution of the extract in the reaction mixture, 

*d* is the thickness of the absorbing layer (mm); and

*t* is the reaction time (s).

**Light and electron microscopy**

The bottom of a plastic box (approximate dimensions 40 × 40 × 7 cm) was covered with four gauze layers moistened with a CNM solution or water (in the control sample). 100 *O. arenaria * seeds were placed on the gauze and exposed either to the CNM solution at the concentration 300 mg/l or water without CNM.

After 5 and 10 days of exposure to CNM, the seedlings were fixed for light and electron microscopic studies . For light microscopy, the seedlings were fixed in a 3:1 mixture of 96% ethanol and acetic acid for 16 h. After fixation, the plant samples were put into 70% ethanol. The plant parts under study (roots, leaves, coleoptiles) were then placed onto a glass slide into a drop of 45% acetic acid. The preparations of whole mount plant parts were made according to the standard procedure [17]. The preparations were analyzed using a Leica DM1000 light microscope (objectives ×10, ×20, ×40, and ×100). The images were recorded with a Leica DFC 295 digital camera (sensor size 3 × 10 ^6 ^ pixels).

For transmission electron microscopy (TEM), the seedlings were fixed with 2.5% glutaraldehyde on a 0.1 M Na-K-phosphate buffer (pH 7.2) supplemented with sucrose (15 mg/ml). The samples were then dehydrated in a series of solutions of increasing ethanol concentrations and embedded in Epon 812, according to the standard procedure. For optimization of MWNTs detection within plant tissues, we opted not to use additional fixation with OsO _4_ and staining with uranyl acetate and lead citrate.

 The sample of pure CNM was prepared for TEM as follows: 25 mg of Taunit was placed onto the surface of unpolymerized resin (Epon 812), poured into a tube. Then, the sample was centrifugated for 3 min at 6,000 g and polymerized, according to the standard procedure. 

**Fig. 1:**
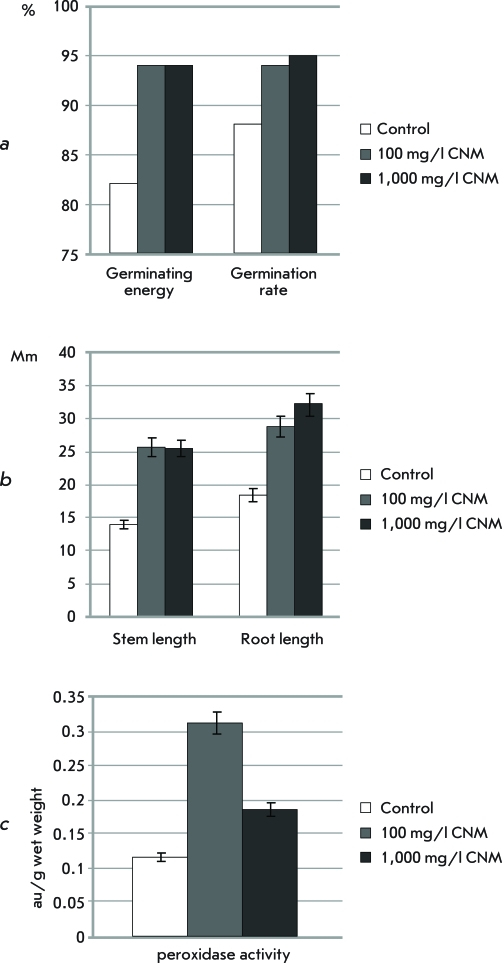
* . * The effect of different concentrations of CNM on the viability, morphological and biochemical parameters of *O. arenaria* seedlings.

 Ultrathin sections of the samples embedded into Epon were investigated by TEM and SAED (selected area electron diffraction) using transmission electron microscopes JEM-1011 (JEOL) equipped with a GATAN ES500W digital camera, and LEO 912АВ (Carl Zeiss). 

##  RESULTS AND DISCUSSION 

**Effect of Taunit on the morphometric and biochemical characteristics of**

*O. arenaria* **seedlings**

In order to characterize the phytotoxicity of CNM, we used seed germination tests, in which the germination energy, germination rate, length of roots and stems, and peroxidase activity were estimated [18]. The germination of *O. arenaria * seeds in the presence of the colloidal solution of CNM Taunit increased the germination energy by 14% as compared with the control sample. CNM with a concentration of 100 and 1,000 mg/l increased the seeds germination rate by 2 and 7%, respectively ( *Fig. 1a* ). Taunit also stimulated the growth of roots and stems of seedlings . At CNM concentrations of 100 and 1,000 mg/l, the root length increased by 55 and 73%, respectively; the length of seedling stems increased by 84 and 82%, as compared with the control sample ( *Fig. 1b* ). Thus, CNM Taunit slightly increased the germination rate and the germination energy of seeds and considerably increased the length of roots and stems of seedlings . Taunit at the concentrations of 100 and 1,000 mg/l also enhanced peroxidase activity in *O. arenaria* seedlings, respectively, to 0.31 ± 0.01 and 0.19 ± 0.02 au/g fresh weight, which is significantly higher than the control value (0.12 ± 0.01) ( *Fig. 1c* ). It is well-known that plants respond to mechanical stress and injury by changing their morphology or growth rate. This phenomenon has become known as thigmomorphogenesis. Thigmomorphogenetic changes are regarded as the adaptation process to stress in plants, and plant hormones play an important role in this process [19]. Under mechanical stress or after injury, the activity of the plants stress hormone, jasmonic acid, increases, whereas the activity of auxin , which controls the processes of morphogenesis and plant growth, decreases. These changes of plant hormone levels may be associated with the increase of peroxidase activity [20-22]. Peroxidases are involved in a number of biological processes, such as photosynthesis, respiration, and protein metabolism. It is an antioxidant enzyme with high sensitivity towards external factors, and this allows using peroxidase activitiy assay for testing of the physiological state of plants. In most cases, a high level of peroxidase activity demonstrates the initiation of the mechanism of a nonspecific response of the plant to stress [23]. It can be assumed that increase in peroxidase activity is associated with the oxidative stress caused by CNM. It has been shown that MWNTs, accumulated at the root surface, often pierce cell walls of epidermal cells [10]. Such interaction can be considered as a mechanical injury and thus elevate the level of peroxidase activity. Our results confirmed that the level of peroxidase activity decreases with an increase in CNM concentration. This observation could be explained by the inactivation of peroxidase molecules by nanotubes due to sorption or other chemical interactions. Our studies demonstrated that, along with an increase in the level of peroxidase activity, exposure to CNM stimulated the growth of roots and stems of plants . Further research is needed to explain the mechanisms of the enhanced growth of plants in the presence of CNM.

**Analysis of CNM Taunit in**

**Fig. 2:**
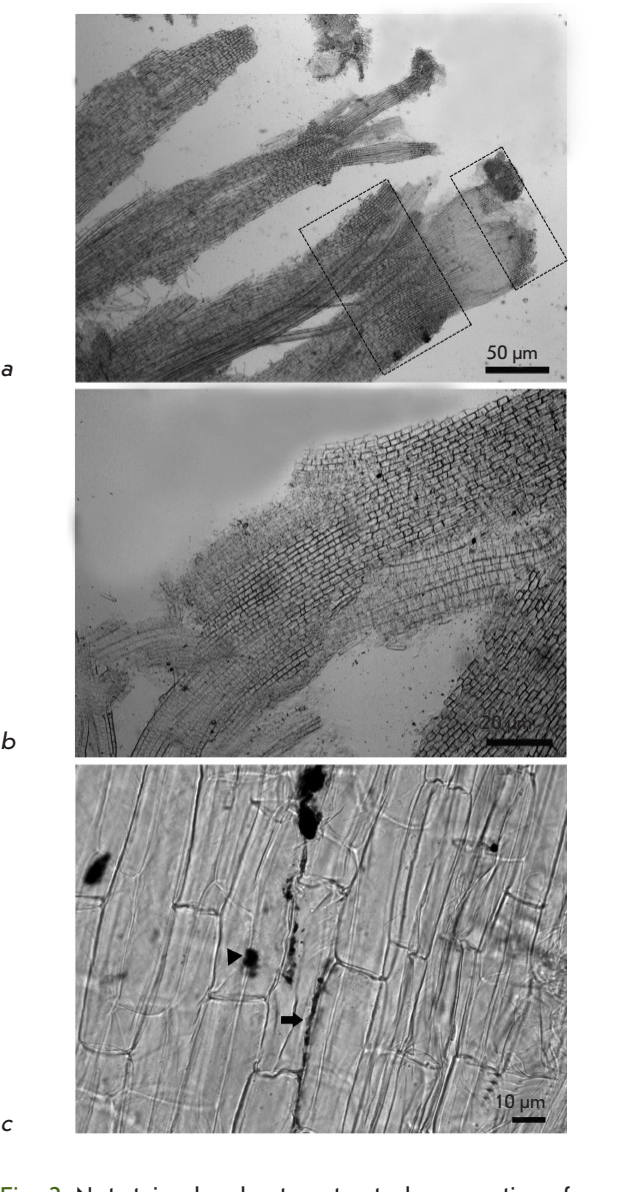
Not stained and not contrasted preparation of a squashed esparcet root. *a* – areas of CNM accumulation in the root are shown by rectangles. CNM decorates root cap, region of maturation and vascular tissues. Scale 50 μm. *b* – inclusions of CNM located in the maturation region. Scale 20 μm. *c* – large accumulations of CNM in the apoplast (arrow) and in the cell (triangular arrow). Scale 10 μm.

**Fig. 3:**
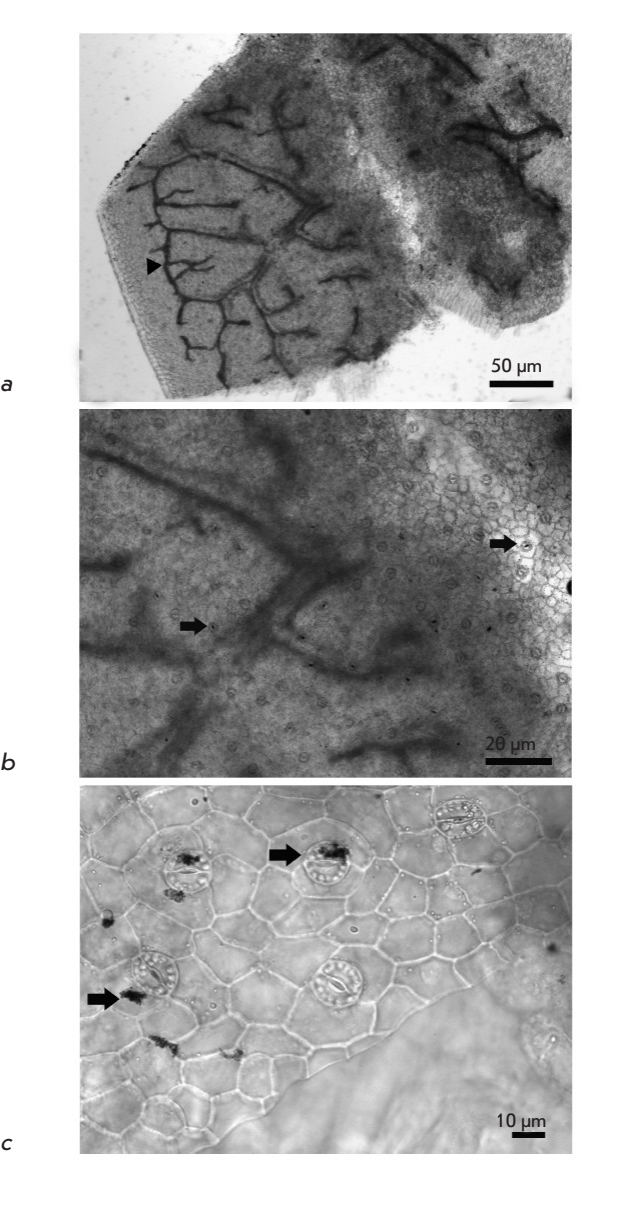
Not stained and not contrasted preparation of a squashed esparcet leaf. *a* – CNM decorates vascular tissue (triangular arrow). Scale 50 μm. *b* – CNM stains vascular strands and localizes in stomata (arrows). Scale 20 μm. *c* – CNM in stomata guard cells and epidermal cells (arrows). Scale 10 μm.

*O. arenaria* **seedlings using light and electron microscopy**

**Fig. 4:**
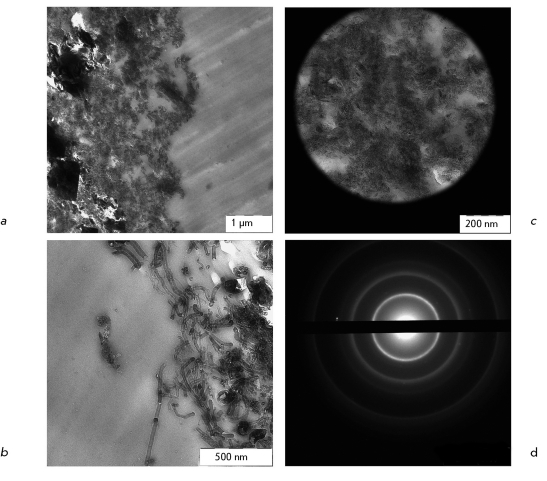
TEM analysis of CNM Taunin sample. *a, b* – ultrathin section of CNM sample; *c* – accumulation of SNM analyzed with SAED; *d* – the diffraction pattern of CNM shown in *c* .

Upon germination of *O. arenaria * in the presence of CNM Taunit, the roots, stems, and leaves of seedlings acquire a characteristic dark gray color. Analysis of whole mount preparations showed that dark gray and black agglomerates are localized on the surface of seedling roots and within particular tissues and cells of the roots, stems, and leaves ( *Figs. 2a-c, 3a-c* ). Ultrathin sections prepared from the plant organs containing aggregations of CNM were analyzed by TEM. Prior to studies of plant material, we had analyzed a pure CNM sample. TEM demonstrated that CNM Taunit ( *Fig. 4a, b* ) contains mostly agglomerates of MWNTs and some inclusions of a nanodispersed electron-dense material (presumably, graphitized carbon). The characteristic features of MWNTs are presented in *Fig. 4b* . The MWNTs without a small amount of inclusions ( *Fig. 4c* ) were characterized by the SAED method . As shown in *Fig. 4d* , due to the regular periodical packing of carbon atoms, MWNTs have an electron diffraction pattern typical for polycrystalline structures. This diffraction pattern was used as a reference sample for the identification of MWNTs in biological material.

**Fig. 5:**
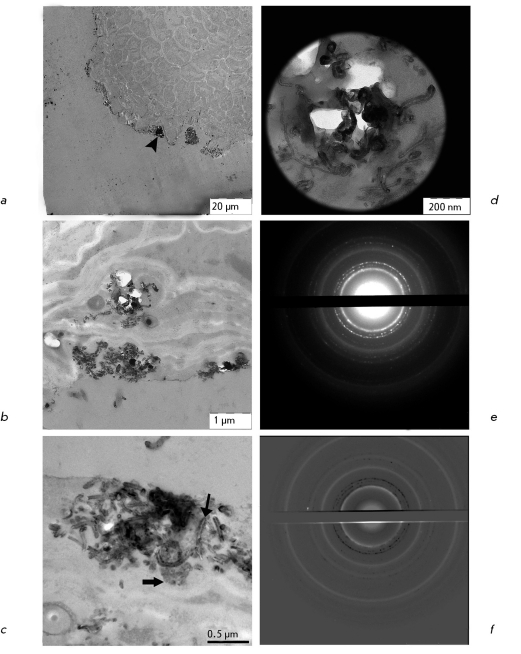
*O. arenaria* seedling grown in the presence of CNM, ultrathin sections of root. *a* – accumulation of CNM on the root surface is shown with arrow; *b* – accumulations of CNM on the root surface and within the root cells; *c* – aggregates of CNM found in the cells containing nanotubes (thin arrow) and finely dispersed electron-dense material (thick arrow); *d* – the area of the root with CNM selected for analysis with SAED; *e* – diffraction pattern of the area shown in *d* ; *f* – superimposed image of pure CNM diffraction pattern ( *Fig* .  *4d* ) and inverted image of the diffraction pattern of CNM found in the root cell ( *Fig.*   *5e* ). The overlapping diffraction spots are white; non-overlapping – black.

**Fig. 6:**
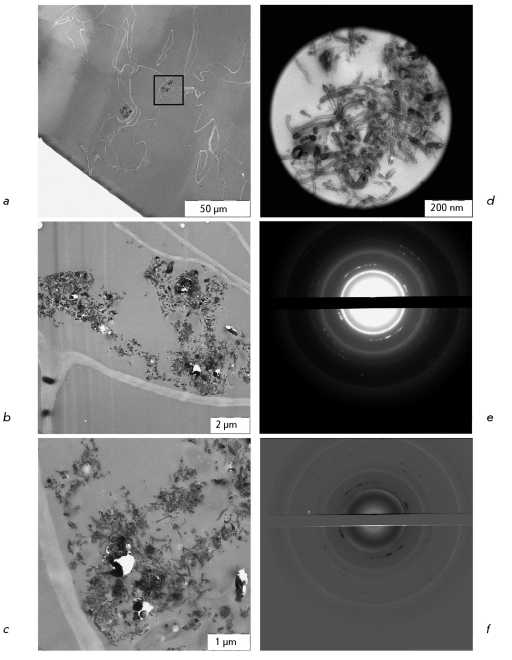
*O. arenaria* seedling grown in the presence of CNM, ultrathin sections of leaf. *a* – the leaf area containing two aggregates of CNM. Framed area is magnified and shown in *b* and *c;* *b, c * – CNM contains nanotubes and finely dispersed electron dense inclusions; *d* – the area of the leaf with CNM selected for analysis with SAED; *e* – diffraction pattern of the area shown in *d* ; *f* – superimposed image of the pure CNM diffraction pattern ( *Fig* . *4d* ) and inverted image of the the diffraction pattern of CNM found in the leaf cell ( *Fig.*   *6e* ). The overlapping diffraction spots are white; nonoverlapping – black.

The analysis of ultrathin sections of roots and leaves showed that CNM is present on the surface and inside the seedling organs. The adsorption of MWNTs on the root surface ( *Fig. 5a* ) has also been reported by other researchers [3, 10, 11]. Furthermore, agglomerates and single MWNT of different lengths are detected in root ( *Figs. 5b,c* ) and leaf cells ( *Figs. 6a-c* ). Thus the MWNTs are unmistakably identified by TEM. However, if MWNTs are located transversely or at some angle to the section plane, it is difficult to distinguish the MWNT fragments from different electron-dense endogenous inclusions. The method SAED can be used to identify MWNT in plant tissues ( *Figs. 5d, 6d* ). Superposition of electron diffraction patterns of MWNTs found in biological samples ( *Fig. 5e, 6e* ) with the reference electron diffraction pattern ( *Fig. 4d* ) confirms the presence of MWNTs in plant cells. Additional diffraction spots on the electron diffraction pattern are accounted for by the presence of endogenous crystalline inclusions in plant tissues.

The accumulation of MWNTs on the root surface was reported by many authors [3, 9–11] and it has been suggested that interaction of MWNTs with plant organs affects plant growth and development [13]. Adsorption of a large amount of MWNTs on the root surface may suppress the water flux and uptake of nutrients, thus inhibiting plant growth [11]. Conversely, the stimulation of seed germination may be associated with the fact that nanotubes (SWNTs) pierce the seed cover and increase water uptake, facilitating seed germination and plant growth [9]. However, SWNTs not only pierce the cell wall , but also penetrate inside the cells; this phenomenon was demonstrated using FITC-labeled SWNTs (SWNT/FITC) [24]. Insertion of MWNT into the wall of epidermal cells and root hairs up to 4 µm was observed in wheat seedlings using two-photon excitation microscopy; however, penetration of whole MWNTs into the cytoplasm was not noted [10]. The authors assumed that penetration, uptake and accumulation of MWNTs are less evident due to the larger nanotube diameter as compared with that of SWNT [10].

##  CONCLUSIONS 

We demonstrated that MWNTs penetrate cell walls, accumulate in the cells and tissues, and most likely are transported via a plant’s vascular system from roots to stems and the leaves of *O. arenaria* seedlings. We argue that the stimulation of *O. arenaria * roots and stems growth and the increase in peroxidase activity were induced by the oxidative stress which develops due to the accumulation of MWNTs in plant cells and tissues.
